# Tea intake and lung diseases: a Mendelian randomization study

**DOI:** 10.3389/fimmu.2024.1328933

**Published:** 2024-02-05

**Authors:** Zhengyan Wu, Min Jiao, Chenying Shu, Chang Li, Yehan Zhu

**Affiliations:** ^1^ Department of Health Management Center, The First Affiliated Hospital of Soochow University, Suzhou, China; ^2^ Department of Pulmonary and Critical Care Medicine, The First Affiliated Hospital of Soochow University, Suzhou, China; ^3^ College of Pharmaceutical Sciences, Soochow University, Suzhou, China; ^4^ Department of Pulmonary and Critical Care Medicine, Chongzhou People’s Hospital, Chongzhou, China

**Keywords:** standard tea intake, black tea intake, green tea intake, lung diseases, squamous cell lung cancer, Mendelian randomization, causal relationship

## Abstract

**Background:**

Existing studies on the relationship between tea intake and lung diseases have yielded inconsistent results, leading to an ongoing dispute on this issue. The impact of tea consumption on the respiratory system remained elucidating.

**Materials and methods:**

We conducted a two-sample Mendelian randomization (MR) study to evaluate the associations between five distinct tea intake phenotypes and 15 different respiratory outcomes using open Genome-wide association study (GWAS) data. The inverse variance weighted (IVW) was used for preliminary screening and a variety of complementary methods were used as sensitivity analysis to validate the robustness of MR estimates. Pathway enrichment analysis was used to explore possible mechanisms.

**Results:**

IVW found evidence for a causal effect of standard tea intake on an increased risk of lung squamous cell cancer (LSCC) (OR = 1.004; 95% CI = 1.001–1.007; P = 0.00299). No heterogeneity or pleiotropy was detected. After adjustment for potential mediators, including smoking, educational attainment, and time spent watching television, the association was still robust in multivariable MR. KEGG and GO enrichment predicted proliferation and activation of B lymphocytes may play a role in this causal relation. No causalities were observed when evaluating the effect of other kinds of tea intake on various pulmonary diseases.

**Conclusion:**

Our MR estimates provide causal evidence of the independent effect of standard tea intake (black tea intake) on LSCC, which may be mediated by B lymphocytes. The results implied that the population preferring black tea intake should be wary of a higher risk of LSCC.

## Introduction

According to data published by the Global Burden of Disease (GBD), Injuries, and Risk Factors Study, respiratory diseases were responsible for the fourth position in terms of leading causes of death (lower respiratory infections), the sixth position (COPD), the twelfth position (tuberculosis), and the seventeenth position (lung cancer) among people of all ages worldwide (1). In 2017, chronic respiratory disease affected approximately 544.9 million individuals globally, marking a 39.8 percent rise compared to 1990, as reported by the chronic respiratory disease collaborators of GBD (2). Between 2010 and 2019, there was a global rise in the incidence and death rates of lung cancer (3). These statistics clearly demonstrate the widespread prevalence of pulmonary ailments and their significant impact on the quality of human life. Therefore, understanding the causes of these respiratory diseases is crucial in order to minimize their occurrence. While the risk factors or beneficial factors of respiratory system diseases are not completely clear.

Tea is widely consumed across the globe for its numerous health advantages such as cancer prevention, protection against heart disease and diabetes (4), and the potential to reduce the risk of all-cause mortality (5–7). Sometimes tea is used for medical care for COVID-19 (8), particulate matter-induced lung injury (9), and acute pancreatitis-induced lung injury (10). Previous research has demonstrated that drinking tea can provide a safeguard against the development of lung cancer, as indicated by multiple studies (11, 12). Conversely, certain studies have failed to establish a correlation between tea consumption and the occurrence of lung cancer (13–16). Additionally, a few other studies have suggested that tea consumption might increase the risk of developing lung cancer (17, 18). Taken together, the association between tea consumption and pulmonary diseases remains equivocal, partially due to the constraints of conventional observational studies, including potential reverse causality, and confounding factors.

Using genetic variations as instrumental variables (IVs), Mendelian randomization (MR) analysis investigates the causal relationship between exposure and disease outcome (19). Benefiting from publicly available genome-wide association study (GWAS) data reporting the genetic effect of single nucleotide polymorphisms (SNPs) on a variety of phenotypes, MR has been widely carried out for causality inference. Given that genetic variants are randomly assigned at conception and are typically free from environmental influence, MR is less influenced by residual confounding and reverse causation. Therefore, in this study, we aimed to utilize the MR technique to evaluate the potential impact of tea intake on respiratory diseases.

## Materials and methods

### Study design

In our study, we utilized a two-sample MR approach to examine the potential causal relationship between tea intake and respiratory diseases. Considering different kinds of tea intake might exert various effects on health outcomes, we used several tea intake phenotypes for our MR analysis, including tea intake, green tea intake, herbal tea intake, standard tea intake, and decaffeinated tea intake. For respiratory outcomes, totally 15 phenotypes were included, which could be grouped into 5 categories, including lung cancer (overall lung cancer, lung adenocarcinoma, squamous cell lung cancer, and small cell lung carcinoma), infectious pulmonary disease (bacterial pneumonia, viral pneumonia, pneumonia, and respiratory tuberculosis), airway disorders [asthma, bronchiectasis, chronic obstructive pulmonary disease (COPD)], lung function indexes [forced vital capacity (FVC), and forced expiratory volume in 1 second/FVC (FEV1/FVC)], and additional respiratory conditions [idiopathic pulmonary fibrosis (IPF), and pulmonary embolism].

**Figure 1:**
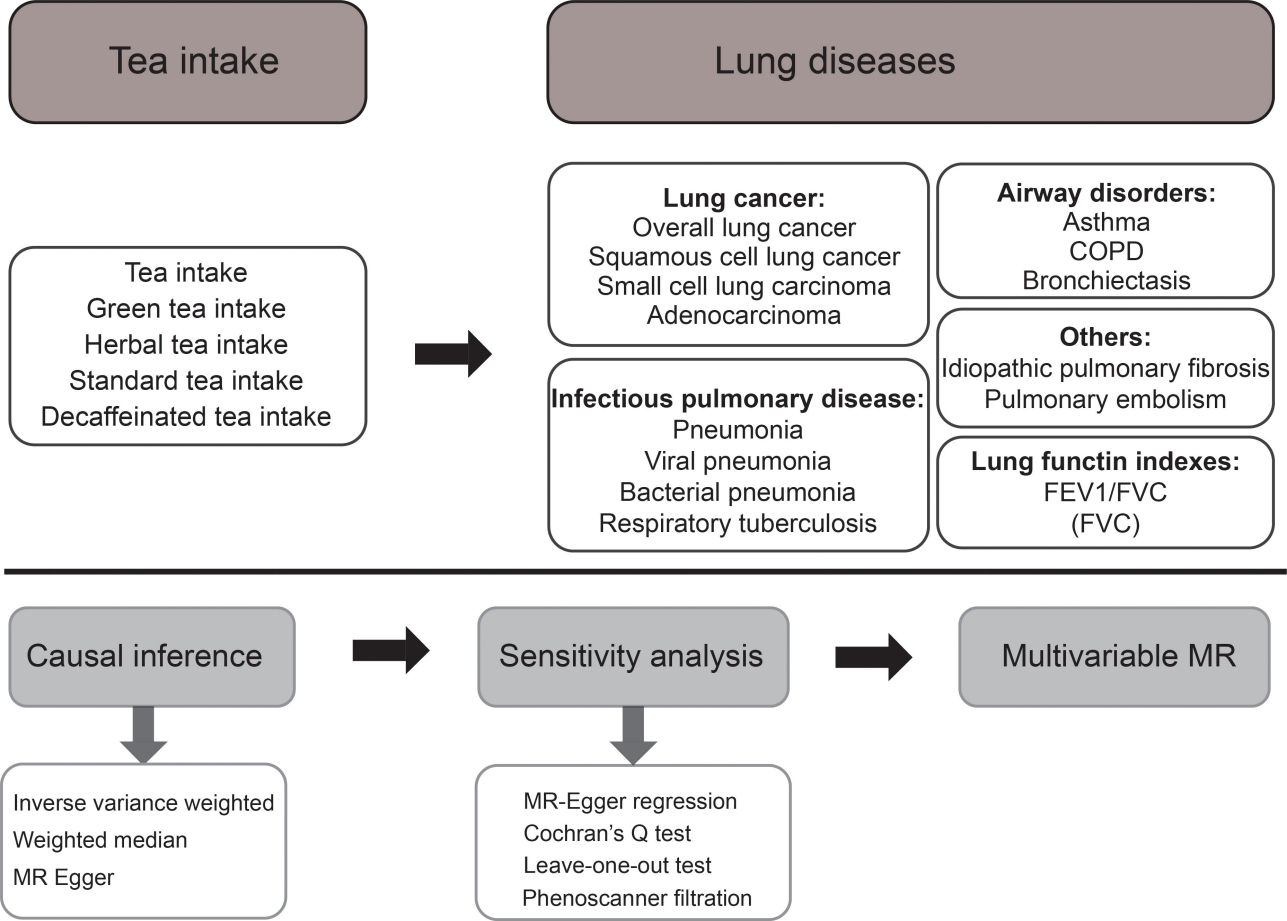
The MR framework of ideation for our study.

The conceptual MR framework is presented in **Figure 1**. The MR design is based on three key assumptions: (A) Single-nucleotide polymorphism (SNPs) are closely related to tea intake exposure; (B) SNPs are uncorrelated with known confounding factors; (C) SNPs affect respiratory outcomes only through tea intake exposure (19). To meet the assumptions presented above, we used a series of instruments quality control steps, and complementary statistical methods under the MR framework. All the analyses were performed depending on the R program (version 4.0.0) utilizing the “TwoSampleMR” package (version 0.5.4) and the “MendelianRandomization” package (version 0.5.1).

### Data source

#### Exposures

The GWAS data for five tea intake phenotypes came from the United Kingdom biobank (UKB) (20), which could be retrieved from the IEU Open GWAS website (https://gwas.mrcieu.ac.uk/) using distinct GWAS ID (**Table 1**). Among them, tea intake encompasses the intake of both green tea and black tea. Standard tea intake encompasses all kinds of tea produced using black tea leaves (which is the most prevalent form of tea), including teabags, loose-leaf tea, and decaffeinated types. Essentially, standard tea intake is the representative intake of black tea.

**Table 1:** Detailed information for the public data.

	ID	Trait	Ncase	Ncontrol
Exposure	ukb-b-6066	Tea intake	447,485	-
	ukb-b-4078	Green tea intake	64,949	-
	ukb-b-13344	Herbal tea intake	64,949	-
	ukb-b-3291	Standard tea intake	64,949	-
	ukb-b-8553	Decaffeinated tea intake	64,949	-
Outcome	ieu-a-987	TRICL-Lung cancer	29863	55586
	ieu-a-984	TRICL- lung adenocarcinoma	11,245	54,619
	ieu-a-989	TRICL-Squamous cell lung cancer	7,704	54,763
	ieu-a-988	TRICL-Small cell lung carcinoma	2,791	20,580
	ebi-a-GCST90014325	Asthma	56,167	352,255
	ebi-a-GCST007431	Lung function (FEV1/FVC)	321,047	-
	ebi-a-GCST007429	Lung function (FVC)	321,047	-
	finn-r9	COPD	18,266	311,286
	finn-r9	Bronchiectasis	2,188	311,286
	finn-r9	Idiopathic pulmonary fibrosis	2,018	373064
	finn-r9	Pulmonary embolism	9,243	367,108
	finn-r9	Pneumonia	58,174	319,103
	finn-r9	Viral pneumonia	3,394	314,673
	finn-r9	Bacterial pneumonia	16,244	314,673
	finn-r9	Respiratory tuberculosis	1,793	374,922

#### Outcomes

The GWAS data for lung cancer as well as subtypes came from the Transdisciplinary Research in Cancer of the Lung (TRICL) consortium (21), which could be obtained from the IEU consortium. For infectious pulmonary diseases and additional respiratory conditions, the GWAS data for all the phenotypes were obtained from the FinnGen consortium (Round 9, website: https://www.finngen.fi/fi). The FinnGen study is a global project consisting of nearly 50,0000 participants, and all the endpoints follow the treelike subtyping system of the ICD-10 classification systems (22). For airway disorders, except for bronchiectasis and COPD coming from the FinnGen study, the genetic information for asthma came from the GWAS data conducted by Valette et al (23). Finally, we also obtained the GWAS data for lung function indexes from the study conducted by Shrine et al (24). To avoid sample overlapping that would bias the MR estimates, the outcome phenotypes included in this study did not contain UKB participants. Detailed information for the GWAS data used in this study is presented in **Table 1**.

### Quality control of instrumental variables

We performed IVs quality control to filtrate eligible SNPs based on the following steps (1): For tea intake, green tea intake, and herbal tea intake, SNPs significantly associated with them at the genome-wide significance level (P< 5 × 10^-8^) were extracted. For standard tea intake and decaffeinated tea intake, we extracted SNPs at a P threshold of 5 × 10^-6^ due to a restricted number of SNPs that reached genome-wide significance. (2) To make sure SNPs were unrelated to one another, we pruned SNPs at linkage disequilibrium (LD) r^2^< 0.001 within a range of 10000kb (25). (3) The F-statistic was then calculated to evaluate the strength of each instrument using the formula $F=\frac{\left(N-2\right)\times R^{2}}{\left(1-R^{2}\right)}$ , of which R^2^ represents the proportion of variance in phenotype explained by a single SNP, which could be calculated using the formula $R^{2}=\frac{2\times Beta^{2}\times EAF\times \left(1-EAF\right)}{2\times Beta^{2}\times EAF\times \left(1-EAF\right)+2\times SE^{2}\times EAF\times \left(1-EAF\right)\times N}$ (26) where EAF represents the effect allele frequency, Beta represents the estimated genetic effect of the SNPs on exposure, N represents the sample size, and SE represents the standard error of Beta. SNPs with an F-statistic of less than 10 were identified as weak IVs and were eliminated (27). (4) We then extracted the SNPs from the outcome data and removed those tightly associated with the outcome phenotypes (P< 5 × 10^-6^), and if the SNPs were not found in the outcome data, we attempted to search and use proxies at LD r^2^ > 0.8 in priority, and for those without suitable proxies were finally discarded. (5) To ensure that the genetic associations reflect the same effect allele, we harmonized the exposure and outcome dataset and eliminated SNPs with incompatible alleles or palindrome structures whose sequence direction was not determined. (6) Finally, before performing MR analysis, we conducted MR- Pleiotropy Residual Sum and Outlier (MR-PRESSO) to recognize and eliminate SNPs with potential pleiotropy (28). After implementing the aforementioned procedures, the remaining SNPs were ultimately determined as eligible IVs to assess the causal impact of various tea consumption on respiratory diseases.

### Univariable MR analysis

We conducted a primary MR analysis using the random-effects inverse variance weighted (IVW) method to initially evaluate the causal association between distinct tea intake and various respiratory diseases. By utilizing a meta-analysis approach, this technique combines the pooled causal effect of the exposure on the outcome, derived from the Wald ratio causal estimates obtained from each of the SNPs. Besides, The random-effects IVW method yields a more cautious causal inference by considering the uncertainty caused by pleiotropy, in contrast to the standard fixed-effects IVW approach (29). The random-effects IVW is referred to as ‘IVW’ unless stated otherwise in this work. For the IVW estimates, Bonferroni correction was applied to account for multiple tests, and an observed estimate with P< 0.0033 (0.05/15 outcomes) was recognized as statistically significant.

### Sensitivity analysis

To examine potential violations of the second and third hypotheses of MR, various methods, including the weighted median and MR-Egger regression were employed (30). The weighted median is the method that yields more conservative estimates as it assumes less than 50% of IVs are invalid (30). The MR-Egger is the statistical method with weak power and is typically used for direction validation.

To detect heterogeneity, Cochrane’s Q test was conducted, and the Q-value with P< 0.05 was deemed as heterogeneity detected. Horizontal pleiotropy was then assessed based on the evaluation of the MR-Egger intercepts. Specifically, the intercept term with P< 0.05 indicated the existence of horizontal pleiotropy, which would bias the MR estimates (31). Furthermore, a sensitivity analysis, called “leave-one-out”, was conducted by leaving out one SNP at a time to assess if a single SNP had a disproportionate effect on the overall estimation. Finally, we searched on the PhenoScanner3 website (http://www.phenoscanner.medschl.cam.ac.uk) to investigate the presence of SNPs linked to any confounding factors, including smoking behaviors (32–34), sedentary behaviors and physical activity (35, 36), educational attainment (37), anti-oxidants (38), dietary intake and food supplement intake (39), and job of participants (40), which have been identified as common risk factors of respiratory diseases. If any were found, we eliminated these SNPs and replicated the IVW analysis to evaluate the robustness of the results.

### Multivariable MR analysis

For the identified causal associations, we further used MVMR analysis to distinguish whether the observed association was driven by potential confounders. Previous studies revealed that the mutational burden of human bronchial epithelium is primarily affected by smoking (32), which also leads to lung cancer (33, 34). In an MR study, it was found that having a genetic inclination towards 3.6 more years of education was linked to a 52% decrease in the risk of developing lung cancer (37). A meta-analysis also reported sedentary behavior indexed by television watching was associated with an increased risk of lung cancer (36). Additionally, an MR analysis revealed the causal impact of time spent watching television on both lung cancer and squamous cell lung cancer (35). As such, considering the tight association between smoking, educational attainment, and sedentary television watching with lung cancer, we adjusted these three factors one by one in the MVMR models. The GWAS for the smoking phenotype came from GWAS & Sequencing Consortium of Alcohol and Nicotine use (GSCAN) consortium, comprising 607,291 European descents for smoking initiation (41) The GWAS for educational attainment was conducted by The Social Science Genetic Association Consortium (SSGAC), with 766,345 participants investigated for years of schooling (42). GWAS for time spent watching television was from the UKB cohort, including 437,887 participants. The data were all extracted from the IEU consortium using the GWAS ID of “eu-b-4877”, “ieu-a-1239” and “ukb-b-5192”, respectively for smoking initiation, educational attainment, and television watching.

### Pathway enrichment analysis

To further uncover the possible mechanism of how standard tea intake promoted LSCC progression, we performed pathway enrichment analysis. Initially, we employed the Kyoto Encyclopedia of Genes and Genomes (KEGG) enrichment analyses. Gene list annotation and analysis were conducted in Metascape, a user-friendly online portal available at http://metascape.org/gp/index.html. The analysis and visualization of data were performed using https://www.bioinformatics.com.cn, a freely available online platform for GO Enrichment.

## Results

### Study overview

The present research evaluated the causal impact of five distinct tea intake phenotypes on the risk of 15 respiratory outcomes. After rigorous IVs quality control, the number of SNPs utilized for each exposure-outcome pair ranged from 14 to 40, and the range of F-statistics was between 20.87 and 493.64, indicating no weak IVs were included in our study (**Supplementary File 1**, **Supplementary Table S1-15**). For binary outcomes, the MR estimates were presented as odds ratios (OR), whereas for continuous outcomes, the MR estimates were shown as beta. A 95% confidence interval (CI) was provided.

### UVMR results

**Figure 2:**
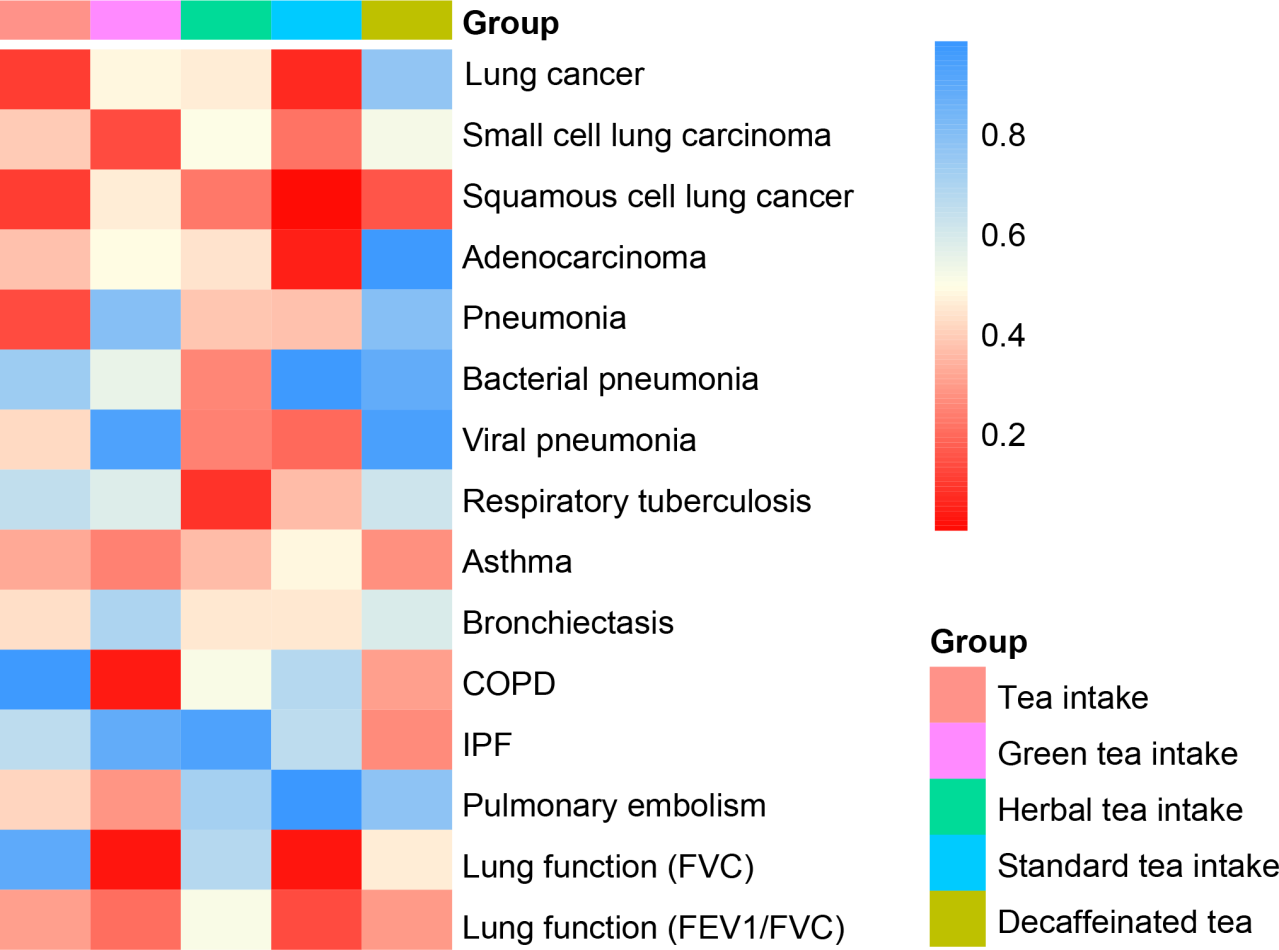
Heat map of causal analysis results for 5 exposures and 15 outcomes, based on *p*-value of IVW results.

**Figure 3:**
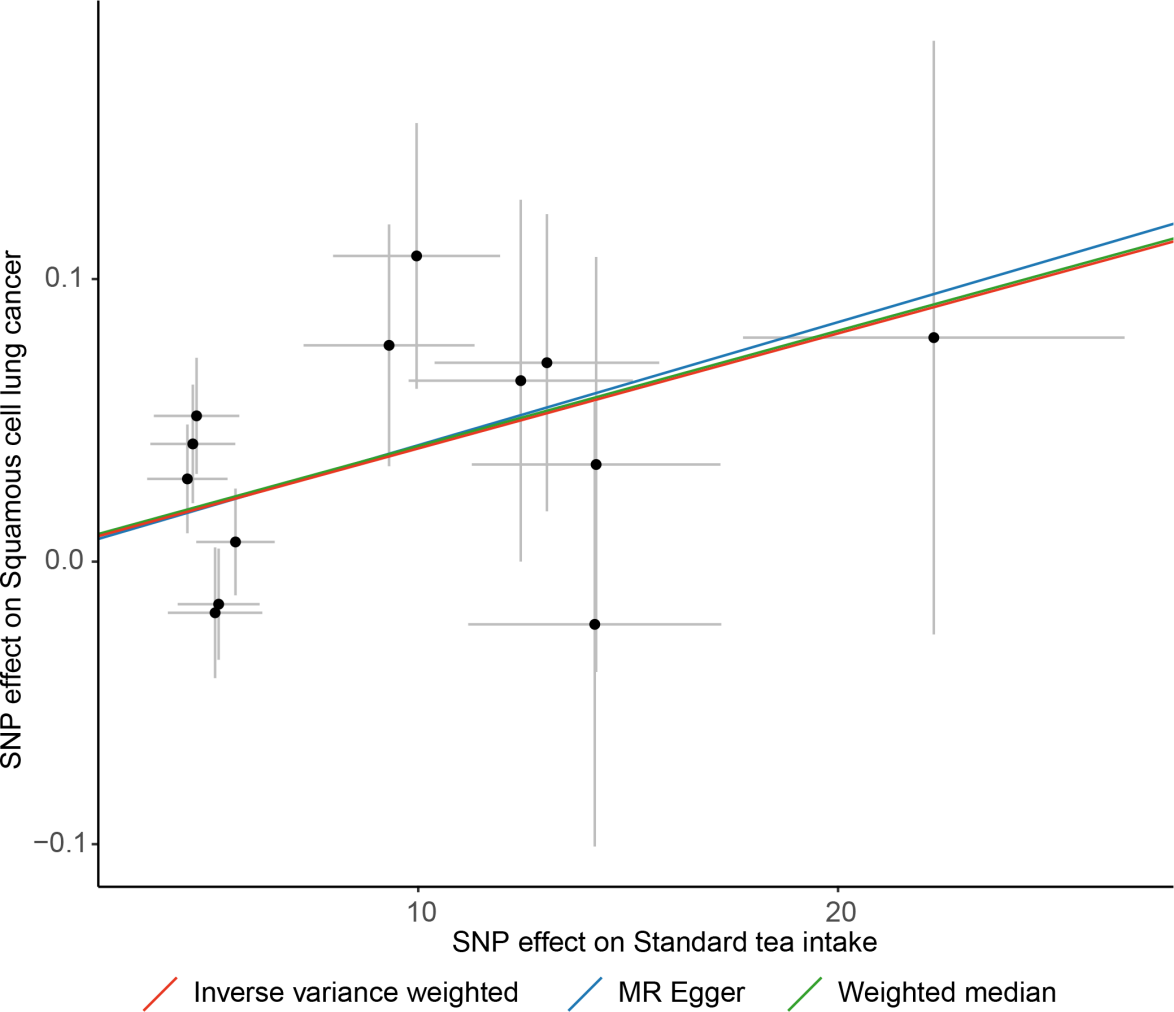
Scatter plot of causal association towards standard tea intake on lung squamous cell cancer.

The IVW method showed that genetic inclination towards standard tea intake was significantly associated with an increased risk of lung squamous cell cancer (LSCC) (OR = 1.004; 95% CI = 1.001–1.007; P = 0.003) (**Figure 2**). The weighted median and MR-Egger yielded similar results (**Figure 3**, **Table 2**). According to Cochran’s Q-test, there was no evidence of heterogeneity in the IVW model (**Table 2**). No evidence of horizontal pleiotropy was found according to the MR-Egger intercept (**Table 2**). Additionally, the leave-one-out analysis verified that the combined IVW estimate was not dependent on any individual SNP (**Supplementary Figure S1**). Besides, by searching on the PhenoSacnner3 website, we found an SNP (rs7233417) related to educational attainment and time spent watching television. After removing this SNP, replicative IVW estimation remained noteworthy potential significant (OR = 1.004; 95% CI = 1.001–1.007; P = 0.009).

**Table 2:** MR results of causality evaluation and sensitivity analysis between standard tea intake and Squamous cell lung cancer.

Methods	Nsnp	Effector	p-value
IVW/OR (95%CI)	13	1.004 (1.001, 1.007)	2.99×10^-3^
Weighted median/OR (95%CI)	13	1.004 (1.000, 1.008)	0.032
MR Egger/OR (95%CI)	13	1.004 (0.997, 1.012)	0.253
Pleiotropy/Egger_intercept	13	-1.96×10^-3^	0.933
Heterogeneity/Q value	13	15.177	0.232
IVW after confounder SNP removal/OR (95%CI)	12	1.004 (1.001, 1.007)	9.10×10^-3^

MR, Mendelian Randomization; IVW, Inverse variance weighted; OR (95%CI), odds ratio with 95% confidence interval.

The IVW also found subtle evidence of several associations between distinct tea consumption and various respiratory outcomes (**Supplementary File 2**, **Supplementary Table S16**). However, none of them passed the examination of sensitivity analysis.

### MVMR results

For the association between standard tea intake and LSCC, we further conducted MVMR to evaluate the direct effect. The MV-IVW showed that after adjusting for smoking initiation (OR = 1.005, 95% CI = 1.003–1.007, P< 0.001), educational attainment (OR = 1.003, 95% CI = 1.001–1.005, P = 0.002), and time spent on television watching (OR = 1.003, 95% CI = 1.001–1.005, P = 0.01), standard tea intake remained a significant effect on LSCC. MV-median and MV-Egger yielded consistent results (**Table 3**). Besides, using the Egger intercept, we found no evidence of pleiotropy in the MVMR models adjusted for smoking initiation (intercept = 0.005, P = 0.15), educational attainment (intercept = 0.002, P = 0.37), or time spent on television watching (intercept = -0.002, P = 0.41).

**Table 3:** MVMR results between standard tea intake and Squamous cell lung cancer.

Traits	Exposure	mv-IVW		mv-Weighted median		mv-Egger	
		OR (95% CI)	p-value	OR (95% CI)	p-value	OR (95% CI)	p-value
1	Standard tea intake	1.005(1.003,1.007)	<0.001	1.005(1.001,1.009)	0.002	1.003(0.999,1.007)	0.085
	Smoking	1.707(1.393,2.094)	<0.001	1.629(1.258,2.110)	<0.001	1.692(1.380,2.075)	<0.001
2	Standard tea intake	1.003(1.001,1.005)	0.002	1.003(1.001,1.005)	0.023	1.002(0.998,1.006)	0.152
	Education	0.488(0.402,0.591)	<0.001	0.528(0.404,0.689)	<0.001	0.486(0.400,0.590)	<0.001
3	Standard tea intake	1.003(1.001,1.005)	0.011	1.003(0.999,1.007)	0.127	1.004(1.000,1.008)	0.015
	Watching TV	2.635(1.767,3.931)	<0.001	1.917(1.150,3.198)	0.013	2.705(1.803,4.058)	<0.001

MVMR, Multivariable Mendelian Randomization; IVW, Inverse variance weighted; OR (95%CI), odds ratio with 95% confidence interval.

### Pathway enrichment analysis

The associated genes used for KEGG analysis can be found in **Supplementary Table S17**. Both KEGG and GO analysis showed that the effect of standard tea intake on LSCC might be through the regulation of B cell proliferation (**Supplementary Figure S2**).

## Discussion

We examined the causal relationship between tea consumption and respiratory illnesses using a two-sample MR analysis. In our study, genetic liability for standard tea intake was associated with an increased risk of LSCC. Further MVMR analysis demonstrated the observed causality was not driven by the common risk factors of lung cancer, and GO enrichment analysis indicated that B cell proliferation and activation may be the underlying mechanisms linking standard tea intake with LSCC. As far as we know, this MR study is the initial assessment of the impact of distinct tea consumption on various respiratory diseases.

Our results were consistent with some previous studies. In a 7-year follow-up cohort study conducted in Japan, it was found that the consumption of green tea had no correlation with the development of lung cancer. The incidence of lung cancer, after adjusting for multiple variables, did not show any significant difference between individuals who consumed 1 cup, 2 cups, 3 cups, or more cups of green tea per day compared to those who consumed less than 1 cup per day. Furthermore, the risk of lung cancer did not appear to decrease for past, current, and passive smokers who consumed green tea (16). In our study, we also discovered that there is no connection between consuming green tea and respiratory diseases, including lung cancer and its subtypes. A majority of laboratory evidence has demonstrated the anti-tumor effect of green tea polyphenols on tumor cell lines and animal models, while epidemiologic studies in humans failed to get consistent findings. This discrepancy might be attributed to a much higher dose in cell lines or animal models compared with dietary human consumption, confounders, and some different mechanisms between vitro and vivo (13). A prospective cohort study of 500,000 Chinese population aged 30-79 years, which followed them for 10.1 years, showed that daily consumers adding tea leaves over 4.0 g/day had elevated risk of all cancers, including lung cancer (HR, 1.31; CI, 1.17–1.46) once all potential confounders have been taken into account (18). According to the Prostate, Lung, Colorectal, and Ovarian Cancer clinical prospective screening trial (PLCO) conducted in the United States, consuming a cup or more of tea daily was linked to a 5% reduction in overall cancer mortality when compared to consuming less than a cup. However, the study did not find any significant decrease in the risk of site-specific cancers, including lung cancer (15).

Our results were also inconsistent with some of the previous studies. A systematic review reevaluated 19 meta-analyses examining the links between tea consumption and 11 various forms of cancer. The findings indicated that consuming tea was connected to a reduced risk of lung cancer, although the level of evidence was weak (11). A study conducted on 63257 individuals from the Singaporean Chinese population revealed that consuming black tea could lead to a decrease in the risk of developing lung cancer. Additionally, women who consumed moderate quantities of green tea had a lower risk of lung cancer (12). Notably, this study solely gathered baseline information regarding tea consumption, which might undergo modifications over time and potentially result in bias. Besides, this study focused on an Asian population, while the data source we obtained was derived from the European population. Additionally, this study did not distinguish the distinct effects of different kinds of tea consumption, including green tea, black tea, decaffeinated tea, and so on. Another research involving 1,177,156 individuals from 17 cohort studies conducted in the United States, China, Japan, Korea, and Singapore revealed that among Asian cohorts, the consumption of at least 2 cups of green tea per day was linked to a greater risk of lung cancer for both current and non-smokers. Additionally, the intake of oolong tea was found to be associated with an elevated risk of lung cancer among non-smokers, whereas the consumption of black tea did not show any such increased risk (17). Nevertheless, it should be noted that these discoveries cannot be considered causal due to the possibility of remaining confounding factors caused by smoking, including secondhand smoke exposure, as well as alterations in the consumption of coffee and tea after enrolling in the study (17). Given that MR analyses are less susceptible to confounding factors or reverse causation, we proceeded with the investigation of the association between tea intake and respiratory illnesses. Considering the potential effects might vary among different tea compositions, our study included various kinds of tea consumption for MR analysis.

There could be certain mechanisms that might elucidate the causality between standard tea intake and LSCC examined from MR. The utilization of insecticide residue in tea farming (43) could potentially account for the elevated risk of cancer. DNA breaks occurred at a dietary concentration because of pyrogallol-related chemicals and tannins from tea (44). One potential mechanism worthy of proposed was the tea composition of standard tea intake in the issue of LSCC risk. As illustrated by the UKB dataset, the consumption of standard tea encompasses various varieties of tea produced using black tea leaves. Theaflavin and thearubigins are formed as oxidized derivatives of black tea catechins during fermentation. Notably, these black tea polyphenols are unique to black tea and are not present in other types of tea (45), This distinction helps explain the mechanism behind the observed association, indicating that only black tea may contribute to the increased risk of this particular type of cancer. Additionally, our KEGG and GO enrichment analysis further showed that regulation of B cell proliferation might mediate the development of LSCC.

B cells are a significant group of immune cells in the adaptive immune system. In squamous cell carcinoma (46), B cells have also been discovered to play a pro-tumoral function through the accumulation of immune complexes including IgG, which would promote the activation of myeloid cells through FcγR and contribute to inflammation. In non-small cell lung cancer (NSCLC), the traditional pathway is partially activated through an IgM-dependent mechanism (47), leading to a negative prognosis (48). B cells are stimulated in mice with developing tumors and generate antibodies that accumulate in the early cancerous lesions, sustaining long-term inflammation by activating Fcγ receptors (FcγR) on innate cells that migrate into the preneoplastic and neoplastic tumor environment (49). Additionally, the complement has been observed to contribute to pro-tumoral effects by promoting antibody-induced chronic inflammation in CMT and TC1 models of lung cancer (47, 50). Multiple studies indicate that B cells play a crucial role in lung cancer, with B cell proliferation observed in 35% of lung cancer cases (51). Furthermore, B cells are present throughout all stages of lung cancer development, with variations observed across clinical stages and histological subtypes (52, 53). In tumor growth and spread, B cells can hinder T cell reactions, especially by generating immunosuppressive cytokines (54). B cell–enriched tertiary lymphoid structures (TLSs) in a model of inflammation-induced hepatocellular carcinoma (HCC) were discovered to function as a sanctuary for tumor progenitor cells and promote the proliferation of cancerous cells through the secretion of lymphotoxin β (55). Some studies have also discovered that B cells have a positive effect on numerous types of cancer. However, the exact mechanism by which B lymphocytes impact cancer is not yet fully understood (56), It is believed that the tumor microenvironment plays a role in determining the balance between the anti-tumor and pro-tumor activities of B cells (57).

The analysis of RNA-seq on individual cells obtained from tumor tissue samples of patients with non-small cell lung cancer (NSCLC) validates the presence of both primary categories of B cells, specifically the naïve-like and plasma-like B lymphocytes. The levels of naïve-like B cells are reduced in advanced NSCLC, and this decrease is linked to a negative prognosis. When naïve-like B cells from NSCLC patients were co-cultured with two lung cancer cell lines, it was observed that the growth of lung cancer cells was suppressed by the secretion of the factors that negatively regulate cell growth. Additionally, researchers proved that the plasma-like B cells impede the progression of cancer cells during the initial phase of NSCLC, while fostering cell proliferation during the later stages of NSCLC (58). Researchers analyzed a group of 108 individuals with lung squamous cell lung cancer (LSCC) through various techniques such as DNA copy number analysis, somatic mutation examination, RNA-sequencing, and expression proteomics. This comprehensive approach led to the identification of three distinct proteomic subtypes in which the Inflamed and Redox subtypes accounted for 87% of the tumors. The Inflamed subtype, in particular, exhibited a higher presence of B-cells (59). In addition to tertiary lymphoid structures, a considerable number of immune-dense regions lacking germinal center-like structures can be seen in NSCLC. Analysis of transcriptomic data and digital pathology images from nine hundred and thirty-five lung cancer patients revealed that a high intratumoral immune hotspot score, indicating the percentage of immune hotspots interacting with tumor islands, was associated with unfavorable overall survival in LSCC but not in lung adenocarcinoma. LSCC exhibiting elevated intratumoral immune hotspot scores demonstrated a consistent increase in B-cell signatures (60). These findings may indicate a positive relationship between B cells and LSCC.

The current study exhibits several strengths. MR is less prone to residual confounding and reverse causation. Genetic variants are assigned at conception, making them independent of the disease development process. Besides, MR often leverages data from large-scale GWAS, maximizing the utility of existing genetic information. And the results obtained from the current study have more statistical power. In addition, the GWAS data used in this study were all derived from participants of European ancestry, reducing the likelihood of population stratification biasing the MR estimates. Furthermore, using the MVMR technique, our study further distinguished the direct effect of black tea intake (indicated by standard tea intake) on the risk of squamous cell lung cancer. Our study has some constraints. As the data was from Europeans, the result derived from our study could not be directly extended to other populations. Besides, information on tea intake was obtained from self-reported questionnaires, which may contain recall bias and may not accurately reflect actual tea consumption. Finally, we mainly analyzed linear correlations and could not rule out the existence of nonlinear correlations in MR analysis.

## Conclusion

Our MR estimates provide causal evidence that black tea intake (indicated by standard tea intake) might increase the risk of squamous cell lung cancer, independent of education attainment, smoking, and television watching. The promotive effect on LSCC of black tea intake and the absent protective effect found on other pulmonary diseases of each kind of tea intake may need to rethink the role of distinct tea consumption on human health, especially for black tea.

## Data availability statement

The original contributions presented in the study are included in the article/**Supplementary Material**, further inquiries can be directed to the corresponding author.

## Author contributions

YZ: Supervision, Writing – review & editing. ZW: Conceptualization, Data curation, Formal analysis, Investigation, Methodology, Project administration, Writing – original draft. MJ: Data curation, Investigation, Writing – original draft. CS: Data curation, Investigation, Writing – original draft. CL: Methodology, Software, Writing – original draft.
