# Supplementary material for: Tea intake and lung diseases: a Mendelian randomization study
Source: Front Immunol. 2024 Feb 5;15:1328933. doi: 10.3389/fimmu.2024.1328933 (PMC10875148; doi:10.3389/fimmu.2024.1328933)
Supplement: Supplementary file 3 [file DataSheet_3.doc]

| SNP | Gene |
| --- | --- |
| rs10493552 | LHX8 |
| rs10801677 | PTPRC |
| rs10969132 | LING02 |
| rs115709030 | TEX36 |
| rs140745793 | TLR4 |
| rs149011033 | ADAM18 |
| rs184776680 | PKHD1 |
| rs2389763 | AHR |
| rs702841 | KANK1 |
| rs7233417 | CELF4 |
| rs729009 | PIK3C3 |
| rs78981082 | ENKUR |
| rs908817 | TMEM233 |

Supplemental Table S17. the closest genes for each lead SNP of standard tea intake on LSCC analyzed by KEGG enrichment
