# Supplementary figures and images for: Tea intake and lung diseases: a Mendelian randomization study
Source: Front Immunol. 2024 Feb 5;15:1328933. doi: 10.3389/fimmu.2024.1328933 (PMC10875148; doi:10.3389/fimmu.2024.1328933)

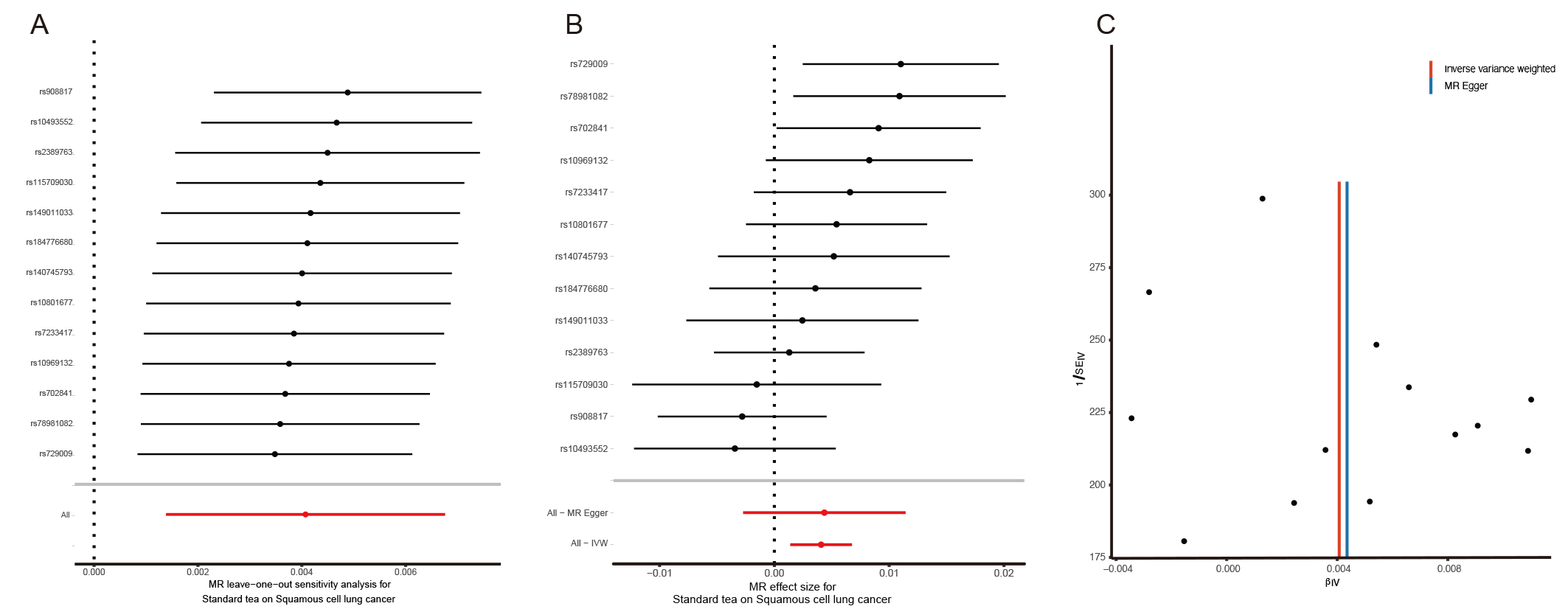

Supplement: Supplementary file 4 [file Image_1.tif]

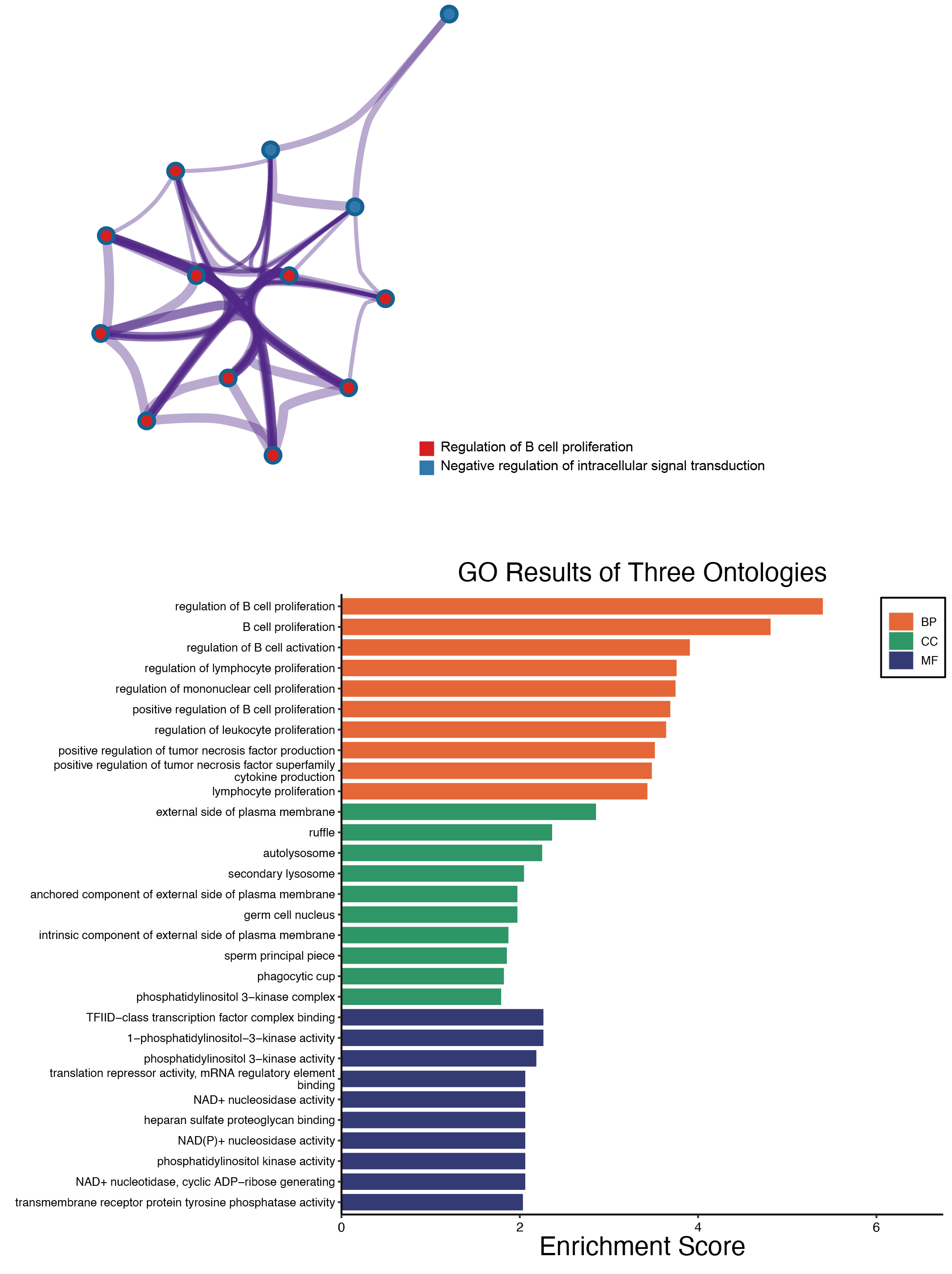

Supplement: Supplementary file 5 [file Image_2.tif]
